# Genetic Manipulation in Mucorales and New Developments to Study Mucormycosis

**DOI:** 10.3390/ijms23073454

**Published:** 2022-03-22

**Authors:** Carlos Lax, José Tomás Cánovas-Márquez, Ghizlane Tahiri, Eusebio Navarro, Victoriano Garre, Francisco Esteban Nicolás

**Affiliations:** Departamento de Genética y Microbiología, Facultad de Biología, Universidad de Murcia, 30100 Murcia, Spain; josetomas.canovas@um.es (J.T.C.-M.); ghizlane.tahiri@um.es (G.T.); sebi@um.es (E.N.); vgarre@um.es (V.G.)

**Keywords:** mucormycosis, virulence, Mucorales, homologous recombination, genetic models, *Mucor lusitanicus*, *Rhizopus microsporus*, CRISPR-Cas9

## Abstract

The study of the Mucoralean fungi physiology is a neglected field that the lack of effective genetic tools has hampered in the past. However, the emerging fungal infection caused by these fungi, known as mucormycosis, has prompted many researchers to study the pathogenic potential of Mucorales. The main reasons for this current attraction to study mucormycosis are its high lethality, the lack of effective antifungal drugs, and its recent increased incidence. The most contemporary example of the emergence character of mucormycosis is the epidemics declared in several Asian countries as a direct consequence of the COVID-19 pandemic. Fortunately, this pressure to understand mucormycosis and develop new treatment strategies has encouraged the blossoming of new genetic techniques and methodologies. This review describes the history of genetic manipulation in Mucorales, highlighting the development of methods and how they allowed the main genetic studies in these fungi. Moreover, we have emphasized the recent development of new genetic models to study mucormycosis, a landmark in the field that will configure future research related to this disease.

## 1. Introduction

Mucorales are a group of early-diverging fungi with many distinct and unique features. One of the most prominent and beautiful features of some Mucorales is their response to light, producing carotenoids and showing a pronounced phototropism [1]. Their unusual and striking reactions to changes in the environment caught the attention of researchers in the beginnings of the first genetic studies. Among those researchers, the Nobel-awarded Max Delbrück dedicated more than 25 years studying the sensory perception in a simple cell using *Phycomyces blakesleeanus* as a model for more complex sensory systems. He believed that this fungus would become an essential model to develop the discipline of molecular biology and understand the interaction of an organism with its environment, the perception of information, the analysis of such information, and its corresponding responses. He also set the trend for many other researchers that continued his work. At that time, many researchers used the classic genetic methodologies (mutagenesis, phenotype selection, and mating analyses) to study the genetic regulation of these fungal responses [2]. However, the advances of this new mucoralean research community were soon hampered by another striking feature of Mucorales: their common reluctance to be genetically transformed [3]. This unconquerable disadvantage motivated many researchers to move their molecular studies to other fungal models with efficient genetic manipulation tools such as *Saccharomyces*, *Candida*, and *Aspergillus* [4,5]. This reluctance to genetic manipulation is responsible for the insufficient knowledge about the order of Mucorales.

The interest to study Mucorales increased at the end of the first decade of this century because of the renewed emergence of the fungal infectious disease known as mucormycosis. Mucormycosis is a lethal disease caused by several mucoralean species, being the most frequent among the genus *Rhizopus*, followed by *Mucor* and *Lichtheimia* (formerly *Absidia*) [6,7,8]. In the past, Mucormycosis was considered a rare infection related to immunosuppressed and otherwise compromised patients. However, new clinical reports and improvements in the correct diagnosis of mucormycosis have shown an emerging increase in the number of cases [9,10]. Indeed, the increased incidence of mucormycosis in COVID-19 patients associated with corticosteroid treatment has raised the scientific and clinical community’s concerns about treating infections caused by the so-named “black fungus” [11,12]. More importantly, some reports also describe an escalating number of mucormycosis cases in healthy patients without known predisposing diseases [13,14].

Furthermore, mucormycosis has mortality rates that can reach up to 90% in the cases of bloodstream disseminated infection [15,16]. These high mortality rates are mainly due to the innate antifungal drug resistance observed in Mucorales, which leaves clinicians with a few poorly effective treatments against mucormycosis [8,17,18,19,20,21]. Besides their natural high antifungal drug resistance, Mucorales can rapidly acquire new antifungal drug resistances through an exclusive RNAi-based mechanism to fast and temporally generate resistant epimutants [22]. In this sense, most of the current studies in Mucorales are focused on investigating new genes, pathways, methodologies, and virulence factors that might be the targets for future antifungal developments against mucormycosis [23,24,25,26,27,28,29,30,31,32]. However, this renewed interest in mucormycosis studies is still hampered by the few modern genetic tools available in Mucorales.

The general reluctance of Mucorales to genetic manipulation has limited the genetic dissection of mucormycosis to the fungal model *Mucor lusitanicus*, previously known as *Mucor circinelloides* f. *lusitanicus* [33]. Homologous recombination was possible only in *M. lusitanicus*, which also allows other genetic tools such as genetic complementation, directed mutagenesis, and tag labeling [23,34,35,36]. These methodologies were used to dissect several genetic mechanisms in Mucorales, including the light responses, the RNAi mechanism, and more recently, the virulence of Mucorales. However, *M. lusitanicus* is not virulent without a strongly immunosuppressed host and an unnaturally high dose of spores in the initial injection [37,38] limitations to genetic studies on pathogenic Mucorales have been recently overcome with a new methodology to transform the fungus *Rhizopus microsporus*, an actual mucormycosis agent frequently isolated from patients [39]. This new genetic model represents a landmark in the current study of mucormycosis and will likely become the leading model in future studies.

**Figure 1:**
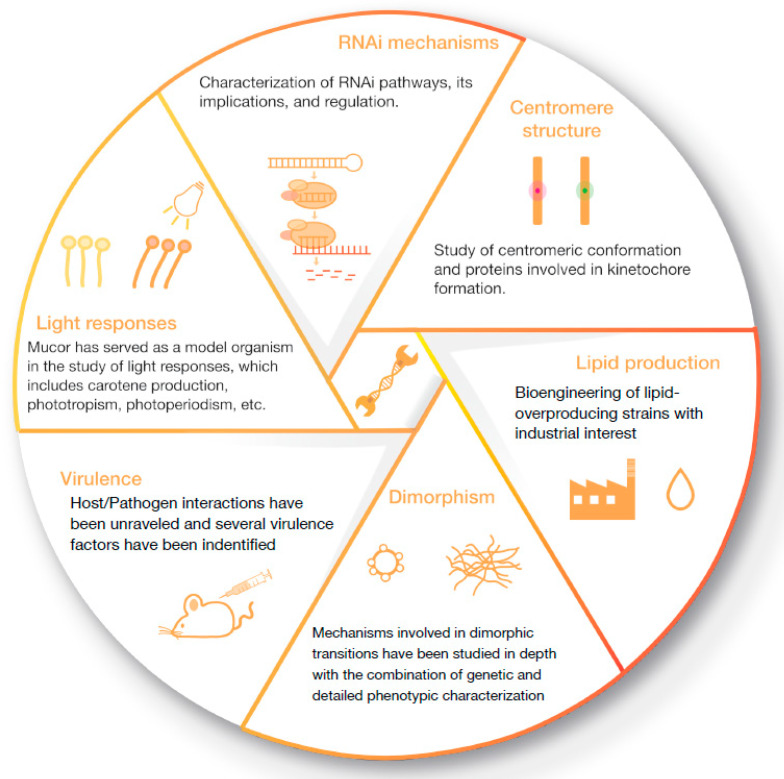
Schematic representation of the leading research fields prompted by the development of new genetic tools in Mucorales.

This review describes the large number of genetic manipulation tools developed in the fungus *M. lusitanicus* and the significant advances achieved through these methodologies (Figure 1). Different attempts to develop genetic models other than *M. lusitanicus* will also be summarized here. Finally, we review the new and promising methods developed in *R. microsporus*.

## 2. *Mucor lusitanicus*, the Primary Genetic Model in Mucorales 

The historical reluctance of Mucorales to genetic manipulation hampered the research of this group of fungi as model organisms. However, they are easily cultured under laboratory conditions and exhibit fast-growing and apparent phenotypes to study many biological processes. The classical model organism of Mucorales was *Phycomyces blakesleeanus*, to which Delbrück dedicated more than two decades studying the interaction of this model organism with the environment. Unfortunately, its inability to be transformed with exogenous DNA forced many researchers to explore other models [40]. The early development of an efficient transformation method of *M. lusitanicus* based on self-replicative plasmids [41] laid the foundation of this fungus as the primary genetic model in Mucorales [42]. This early transformation technique, based on polyethylene glycol (PEG) to allow the DNA entry into protoplast, has been refined all over the years until the successful implementation of the electroporation protocol [43]. Thenceforth, the genetic tools to manipulate the genome of *M. lusitanicus* have grown exponentially, allowing the characterization of the response to light [44,45], RNA interference (RNAi) [46], pathogenesis [23,32], lipids metabolism [47], carotenoids biosynthesis, centromere structure, and dimorphism [36,45,48,49,50] (Figure 1). In this section, we describe the significant number of discoveries that have raised *M. lusitanicus* as a primary fungal model thanks to the early development of the transformation method and the ability of the researchers to exploit it.

### 2.1. Plasmid Transformation, RNAi, and Functional Genomics

*M. lusitanicus* transformation complements auxotrophic markers such as leucine, uracil, and methionine. The obtention of auxotrophs [51] and the characterization of the genes that complement these phenotypes [41,52] allowed their use as selectable markers. Thus, the inclusion of the selectable markers in self-replicative plasmids entailed the development of the first molecular tools in *M. lusitanicus*.

Using these selectable self-replicative plasmids as recipients to construct genomic libraries boosted the characterization of the carotenoid’s biosynthetic pathway. The filamentous fungus *M. lusitanicus* exhibits a yellow phenotype cultured under illumination conditions due to the accumulation of β-carotene as other Mucorales. Before discovering RNAi in *M. lusitanicus*, the implementation of genomic libraries and an accidentally silenced dark-yellow transformant led to the identification of a gene involved in the process of carotenogenesis, the negative regulator *crgA* [49]. This discovery led to the further complete dissection of the silencing mechanism in *M. lusitanicus* [26,53,54,55,56]. The development of a hairpin-RNA expression plasmid permitted the determination of the contribution of each component of the canonical RNAi pathway of *M. lusitanicus.* Therefore, the involvement of the RNA-dependent RNA polymerases (RdRPs) in the canonical RNAi mechanisms of *M. lusitanicus* was unraveled, with RdRP-1 being required to produce the initial dsRNA that triggers the mechanism, and RdRP-2 related to a later feedback loop that amplifies the silencing [57]. Similarly, the expression of hairpin-RNA from plasmids unveiled that Dicer-2 and Ago-1 are the ones involved in the canonical RNAi of this fungus [58].

The thorough characterization of the RNAi machinery accomplished in *M. lusitanicus* led to the development of an elegant strategy of functional genomics to identify virulence factors in this fungus. A RNAi high-throughput library based on silencing plasmids was developed in *M. lusitanicus* to screen and identify virulence factors [23]. The application of this genetic tool allowed the identification of the genes *mcplD* and *mcmyo5,* related to virulence by survival assays first in *Galleria mellonella* infection model and then in mice [23]. Further research based on these screenings will help uncover the complex mechanims that drive fungal infection.

The RNAi mechanism, the carotenogenic pathway, and the publication of the genome (http://genome.jgi.doe.gov/Mucci2/Mucci2.home.html accessed on 21 March 2022 raised the interest of *M. lusitanicus* as a promising model with industrial and scientific interest [50,58,59]. The transformation with plasmids provided a fast method to complement mutated or disrupted genes, demonstrating their implication in the phenotypes observed [35,45,57,60,61]. In addition, the characterization of light-inducible [60] and strong promoters [62] in *M. lusitanicus* permitted the development of new genetic tools to control and overexpress the target genes, respectively. Thus, the overexpression of genes has been successfully applied to improve the production of lipids by increasing the expression of key enzymes involved in the fatty acid biosynthetic pathway [63].

### 2.2. Homologous Recombination and Its Derived Genetic Tools in M. lusitanicus

The use of self-replicative plasmids supposed the beginning of the genetic tools that placed *M. lusitanicus* in a unique position as a model organism. However, the true landmark in the genetics of Mucorales was the development of a methodology to edit the genome of *M. lusitanicus* by homologous recombination [49]. From gene disruption to the most recent protein-tagging strategies, the use of the homologous recombination phenomenon supposed a revolution that converted this fungus into a reference model for genetic manipulation.

The first studies reporting homologous recombination in *M. lusitanicus* appeared right after the development of the transformation method [41,64]. The homologous recombination process supposes the reparation of a double-strand break (DSB) in the DNA by using a similar or identical DNA molecule to replace the region affected [65]. The observation of this process in self-replicative plasmids supposed the first hint indicating that the natural occurrence of DSB was frequent enough for applying it to develop a genome-edition method in *M. lusitanicus* [64,66]. A decade after these first observations, an efficient and directed gene disruption method based on linear DNA fragments was developed [49]. The disruption method consisted of transforming the fungus with a disruption fragment containing a selectable marker flanked by the upstream and downstream sequences of the target gene. After a few vegetative growth cycles under selective conditions to select homokaryon transformants (the protoplasts for the transformation are multinucleated), the selection of stable mutants was easy and fast. Thence, hundreds of genes have been edited, demonstrating their implication in many biological processes.

The first studies that accomplished the genetic manipulation via homologous recombination in *M. lusitanicus* were limited by the absence of genome information. The known sequences derived from the sequencing of plasmids that complemented a mutant phenotype under study [49] or by gene cloning using degenerated primers based on the genes known from other organisms [44,67]. Therefore, the first disruption fragments leverage these known sequences to insert the selectable marker in them. The later publication of the *M. lusitanicus* genome allowed the researchers to know and amplify the target genes’ flanking regions to produce a complete deletion of the ORFs to perform the gene replacement.

The ability to make mutants allowed the characterization of multiple genes related to light response regulation, such as the role of *crgA* and *white collar-1* genes in carotenogenesis and phototropism [44,49]. Then, the RNAi machinery of *M. lusitanicus* was dissected, obtaining null mutants for the *dicer* [60,67], *rdrp* [57,68], and *ago* genes [61]. The analysis of the siRNAs in these null mutants allowed the identification of four classes of endogenous siRNAs, revealing the role of the RNAi machinery to regulate gene expression in *M. lusitanicus* [53,69,70]. Later, the study of this endogenous regulatory mechanism led to the discovery of two novel silencing phenomena, the generation of epimutants [22] and the non-canonical RNAi pathway (NCRIP) [35]. Both RNAi pathways immediately attracted the attention of researchers due to their role in the pathogenic potential of *M. lusitanicus* [29,55,71,72]. After all, the ability of the fungus to generate transient resistance against antifungal drugs [22,73,74], and the identification of NCRIP, a *dicer*-independent silencing mechanism that negatively regulates the generation of epimutants [35,68,75], supposed important implications for antifungal therapies. Finally, the last research field opened in Mucorales facilitated by the virtues of *M. lusitanicus* is the study of the adenine methylation in eukaryotes, a new regulatory mechanism recently discovered in a few ancient eukaryotes for which *M. lusitanicus* represent a unique model [76].

The development of a stable gene replacement method allowed the study of the virulence factors of *M. lusitanicus* beyond RNAi. The deletion of the genes involved in the high-affinity iron uptake mechanism of *M. lusitanicus* demonstrated its involvement in the virulence of this fungus [32]. The expression of this family of iron uptake genes was also related to dimorphism, a critical process previously involved in virulence [48,77,78,79]. The availability of the genome sequence of two different strains of *M. lusitanicus*, the avirulent NRRL 3631 and the virulent strain CBS 277.49, allowed the comparison of the two genomes and the further genetic analysis of the main differential aspects [28]. Similarly, a transcriptomic analysis comparing the avirulent NRRL3631 and the virulent strain CBS 277.49 led to the identification of *wex1,* a new exonuclease involved in virulence [26]. Moreover, transcriptomic analysis during macrophage-spore interaction allowed the identification and further genetic dissection of *atf* transcription factors and their regulatory targets [25].

Overexpression of target genes in *M. lusitanicus* can be achieved by integrating a construction containing this gene under its own promoter or a strong one (for instance, *Pgapdh*) [23,35]. The cassette can be integrated into the *carRP* locus, which is involved in the biosynthesis of carotenoids. The integration in this locus results in albino transformants, which are easily tractable by their visual phenotype [80]. In both cases, the integration by homologous recombination is confirmed by PCR. The construction must also include the upstream and downstream flanking regions of the target gene or *carRP* to replace them with the engineered fragment. Alternatively, self-replicative plasmids can be used for gene overexpression [80]. In this last case, the plasmid must be maintained in the cell by keeping the selective pressure in a minimal medium; otherwise, the segregation of the plasmid is unstable and eventually is lost [34].

Cellular localization of proteins in *M. lusitanicus* is possible due to the development of an efficient labeling tool by fusing specific genes in-frame to the N-terminus or C-terminus with the enhanced green fluorescent protein (eGFP) or the red fluorescent m-Cherry. The construction can be flanked by the promoter and terminator sequences of the target gene and a selectable marker or by a strong promoter when the targets genes are expressed on a low level [36]. In both cases, the integration by homologous recombination is confirmed by PCR. This classic genetic tool widely used in many organisms has been recently implemented in *M. lusitanicus,* allowing the discovery of a particular type of centromeres exclusively found in Mucorales [36].

Additionally, a methodology for functional studies of key aminoacids in proteins was established in *M. lusitanicus*. The strategy is based on producing a construct harboring mutations in the key aminoacids, the unaltered rest of the gene, the marker gene, and the flanking sequences. This construct is expressed in mutant strains lacking the target gene [35].

Finally, the genetic edition by homologous recombination was applied to study the lipid biosynthesis pathways that operate in *M. lusitanicus.* Thus, the role of malate and citrate transporters was elucidated, generating engineered strains with industrial interest [47,63,81,82]. Similarly, recent approaches used overexpression constructs to convert the fungus into a cell factory producing stearidonic acid, dihomo-gamma-linolenic acid, or medium-chain fatty acids [83,84,85].

## 3. *Rhizopus microsporus*, the New Genetic Model to Study Mucormycosis

Among all Mucorales, *R. microsporus* combines several characteristics that make it one of the most interesting species. This fungus is a model for studying symbiotic relationships between fungi and bacteria [86]. While some strains of *R. microsporus* can complete sexual and asexual reproduction independently, others require bacteria to reproduce [86]. Thus, *R. microsporus* is a well-known model of mutualistic symbiosis that may have evolved from a previous antagonistic interaction [87,88]. Moreover, this fungus is also a plant pathogen, causing rice seedling blight [89]. Symbiotic bacteria (*Mycetohabitans* sp., previously classified as *Burkholderia*) produce rhizoxin, a toxin that blocks plant mitosis and allows both fungus and bacteria to live in the necrotized plant tissue [89].

Bacterial presence and rhizoxin production are not essential for developing the lethal disease mucormycosis [90]. Infection caused by *Rhizopus* species supposes around 50% of all cases reported of this disease globally, being the most prevalent genus among all causal fungal agents of mucormycosis [14]. Along with *Mucor*, some mechanisms involved in the pathogenesis of mucormycosis have been unraveled in *Rhizopus*. For instance, macrophages are the first line of defense against the infection, and iron restriction inside the phagosomes regulates host defense [25,91]. In addition, the endothelial CotH proteins are a crucial element in the adhesion and invasion of the tissues [24]. Unfortunately, *Rhizopus* species also present a high reluctance for genetic manipulation, preventing a deeper understanding and characterization of these lethal fungi. The increased recent incidence, the alarming new cases in immunocompetent patients, and the concerning large number of cases associated with COVID-19 are urging for research attention, new models and genetic tools. 

### 3.1. A New R. microsporus Strain for Genetic Transformation: Auxotrophic Isolation and Plasmid Transformation

Undoubtedly, tools that allow for genetic manipulation open many possibilities. Given the limitations and the difficulties associated with genetic modification of Mucorales, a step-by-step optimization of the process was necessary to achieve this complex and longed-for goal. Analogously to previous studies, a uracil auxotrophic strain of *R. microsporus* (ATCC 11559) was isolated [39]. However, unlike previous approaches, the *R. microsporus* auxotrophic strain was isolated spontaneously without mutagenic compounds or UV light [39]. This reduced mutational burden is desirable considering that this strain (UM1) will be used for downstream analysis and characterization, especially virulence assays. This strain carries a non-synonymous substitution in the *pyrF* gene that changes a lysine residue in the active center for a glutamic acid residue (K73E) [39].

The characteristic robust cell wall of Mucorales is one of the main hindrances that hinder genetic manipulation. Thus, proper cell wall digestion with lytic enzymes is critical to ensure efficient transformation [80]. Following the guidelines established for *M. lusitanicus*, it was necessary to select the optimal combination of time and temperature for *R. microsporus* germination and the concentration of lytic enzymes [39]. Initially, the self-replicative plasmid pMAT1819 containing the *pyrF* gene of *R. microsporus* was used for testing different sets of electroporation parameters. With this approach, optimized conditions for the electroporation pulse were established, granting a sufficient efficiency to develop the targeted gene disruption procedure.

### 3.2. Development of a Stable Homologous Recombination Strategy Based on the CRISPR-Cas9 Machinery in R. microsporus

Although plasmid transformation is a relevant landmark itself, the main concern with self-replicative plasmid is that only a variable proportion of descendant spores will carry the plasmid through growth cycles [34].In addition, for applications like RNAi-induced silencing, only descendants with a high copy number of plasmid can trigger silencing mechanisms [34]. The benefits and possibilities that a working genetic modification procedure by homologous recombination can produce have been previously detailed with *M. lusitanicus*. To combine these advantages with the virulent nature of *R. microsporus*, efforts focused on developing an equivalent procedure in this fungus. The successful strategy comprised using in vitro assembled ribonucleoprotein complex by Cas9 and a guide RNA (gRNA) that targets a specific sequence in the genome coupled with DNA templates flanked with micro-homology repair regions (35–40 bp). These short homology regions were adapted from the previously validated strategy developed in other fungi, like *Aspergillus fumigatus* [92]. Interestingly, unlike different methods requiring larger homology regions [93], the CRISPR-Cas9 machinery allows for homologous recombination with much shorter homology regions, making this approach more efficient in terms of experimental time and resources needed.

This strategy was validated using the UM1 strain with the disruption of *leuA* and *crgA* genes, involved in leucine biosynthesis and carotenogenesis repression, respectively [49,94,95,96]. Using two different gRNAs for the disruption of each gene evidenced that differences in Cas9-gRNA in vitro cleavage efficiency correlated with transformation efficiency [39]. Similar to *M. lusitanicus*, *R. microsporus* has multinucleated spores, and several grow cycles in selective media are required to obtain homokaryotic strains. Therefore, only if the integration is stable will it remain through cycles. With this method, in 5-6 cycles of growth, mutant strains were homokaryotic, which is highly desirable for further phenotypic characterization of mutant strains generated and confirms that the integration is stable.

This strategy produced the first visual phenotypes in *Rhizopus* generated with targeted mutagenesis mediated by homologous recombination. In the case of *leuA*, mutants were only able to grow in minimal media when supplemented with leucine, demonstratingthe auxotrophy generated. On the other hand, mutant strains in the *crgA* gene showed a defective development in aerial mycelia and increased melanin levels [39]. Remarkably, this pleiotropic phenotype correlates with the observations made in *M. lusitanicus* [49,95]. The generation of these strains, the development of a reliable procedure, and the examination of these phenotypes represent a promising starting point for future studies of mucormycosis and the biology of *Rhizopus*.

### 3.3. Uracil Auxotrophy Is Directly Related to the Virulence of R. microsporus

As a proof of concept, the wild-type *R. microsporus* strain, the uracil auxotrophic strain (*pyrF*^−^), and the *pyrF* complemented strains (with *pyrF* gene integrated either in *leuA* and *crgA* locus) were tested in mice infection experiments. In contrast with mice infection experiments with *M. lusitanicus,* which require strong immunosuppression and the use of specific mouse strain, *R. microsporus* shows an apparent virulence with immunocompetent Swiss mice [39]. While the wild-type strain of *R. microsporus* killed all mice in the first 6-7 days post-infection, the *pyrF*^−^ strain did not kill any mice, showing an utterly avirulent phenotype. The virulent phenotype was restored in the *leuA* and *crgA* mutant strains when they integrated a functional copy of *pyrF* gene [39]. Consistent with findings in other fungi, uracil autotrophy has also been determined as a virulence trait in *A. fumigatus* and *Candida albicans* [97,98,99]. Studies with *A. fumigatus* revealed that the free uridine/uracil levels present in the host tissues are insufficient for the fungus to grow and develop normally [97]. Considering the virulent nature of *R. microsporus* and the new possibilities that arise with the genetic tools generated, *R. microsporus* is currently positioned as a new reference model organism for further molecular studies in mucormycosis.

## 4. Attempts to Transform Other Mucorales

### 4.1. Homologous Recombination in Rhizopus delemar

*Rhizopus delemar* (previously known as *R. oryzae*) is one of the most frequent causal agents isolated from patients suffering mucormycosis [100]. However, genetic manipulation in *R. delemar*, is quite limited. The aseptate hyphae, the multinucleated vegetative spores, and the duplicated genome are mucoralean features influencing the inefficient generation of stable null mutants [101]. The principal attempt to study a gene function in *R. delemar* was in the high-affinity iron uptake system by gene disruption of one of its components. Iron is an essential micronutrient for all microorganisms, and during infection, pathogenic microbes must obtain it from the host, making it an interesting target for antifungal treatments. In *R. delemar*, the high-affinity iron uptake system has three key elements: an iron reductase (FRE), a ferroxidase (FET3), and a permease (FTR1). A disruption approach was designed more than a decade ago using the auxotrophy marker *pyrF* flanked by two homology fragments for homologous recombination in the *ftr1* locus. The result of this study was an unstable heterokaryon mutant, which was interpreted as evidence of the essential role of this gene in Mucorales. However, further studies demonstrated that this gene could be easily disrupted in *M. lusitanicus* [32]. The absence of new studies trying to perform directed homologous recombination in *R. delemar* highlights the reluctance of this fungus to genetic manipulation.

A more recent study showed that, although homologous recombination is still inefficient in *R. delemar*, point mutation can be directed to target genes using the system CRISPR-Cas9 [102]. The study showed a plasmid containing pmCas9:tRNA-gRNA expressing Cas9 endonuclease and *pyrF*-specific gRNA into two different clinical isolates (FGSC-9543 and CDC-8219). This approach successfully obtained several transformants with a single nucleotide deletion at the CRISPR-Cas9 target site. The same study tried to complement mutations in the *pyrF* gene using a homologous recombining fragment. Again, their results also showed that only ectopically-integrated unstable heterokaryons could be obtained in *R. delemar* [102].

### 4.2. CRISPR-Cas9-Based Mutagenesis in the Fungus Lichtheimia corymbifera

*L. corymbifera* is another causal agent of mucormycosis presenting a high isolation frequency from clinical samples right after *R. delemar* [14]. Like other Mucorales, *L. corymbifera* also strongly resists the traditional genetic manipulation methodologies. The lack of genetic tools has hampered the dissection of the genetic pathways behind the pathogenic potential of *L. corymbifera*. Homologous recombination using exogenous DNA fragments has not been achieved in *L. corymbifera*, not even in an unstable state like in *R. delemar*. However, an adapted methodology based on the CRISPR-Cas9 system and without the necessity of an autoreplicative plasmid worked in *L. corymbifera* to disrupt a target locus [103]. This plasmid-free system directly transformed the *L. corymbifera* protoplasts with the Cas9 protein and two guides RNAs (gRNA) flanking a region of the uracil selective marker gene *pyrG* (encoding the orotidine 5′-phosphate decarboxylase). Uracil auxotrophic strains were selected using 5-fluoroorotic acid (5-FOA) resistance. Protoplasts expressing *pyrG* can convert 5-FOA into 5-fluorouridine monophosphate, a toxic compound that hampers their development [104]. This strategy worked to disrupt an auxotrophy marker gene, and it could work for other selection maker genes with a clear system to differentiate mutants from the rest of the protoplast but not for the rest of genes.

## 5. Conclusions

The genetic manipulation progress in Mucorales has been neglected for a long time due to their reluctance to accept foreign DNA and further genomic integration. However, a broad collection of new genetic methodologies has been developed in the last decade, correlating with a long list of research studies that were not possible before those methods. In this review, we have summarized all the advances in the genetic manipulation of Mucorales that helped in different studies of the mucoralean cell physiology, with a particular focus on the analysis of mucormycosis. The repertory of advanced genetic tools available in *M. lusitanicus* described here makes clear that this fungus is still the most amenable study model for the genetic dissection of most cellular processes in Mucorales. Regarding the pathogenesis of Mucorales, *M. lusitanicus* has been the primary genetic model during the last decade [29]. The first studies linked the size of the spore and the germination velocity with virulence [37]. A genomic platform based on the RNAi mechanism identified new genes involved in virulence [23]. The primary study of the RNAi mechanism in *M. lusitanicus* led to discovering an antifungal drug resistance mechanism conserved only in Mucorales based on the generation of resistant epimutants [22,26,56,68,73,74]. The high-affinity iron uptake system, an essential process in the virulence of most pathogens, was also genetically studied in *M. lusitanicus* [32]. Different genomic and transcriptomic approaches identified gene profiles related to virulence, and many genes from these profiles were mutated and functionally validated in survival assays [25,28]. In addition, the study of the transduction pathways in *M. lusitanicus* led to identifying new genes and pathways related to virulence [79,105]. Thus, *M. lusitanicus* has been an invaluable genetic model in studying genes and pathways associated with the virulence of Mucorales.

However, *M. lusitanicus* shows reduced virulence in the survival assays performed in the laboratory using murine models, and more striking, it has never been isolated from a patient as a causal agent of mucormycosis [37]. The recent development of the methodologies allowing stable homologous recombination in *R. microsporus*, one of the most usual causal agents of mucormycosis, represents a landmark in the study of mucormycosis. This development will make *Rhizopus microsporus* the leading choice for all future studies related to virulence. These recent studies showed the possibility of disrupting genes and later complementing the mutations with two different auxotrophy marker genes. The possibility of performing homologous recombination in *R. microsporus* predicts that other genetic techniques will soon be developed in this fungus, such as directed mutagenesis and aminoacid substitutions, overexpression, tag-labeling, and RNAi. Current and future techniques, the virulent wild type strain (positive control) and the avirulent uracil auxotrophic strain (negative control), constitute the perfect platform to study the pathogenic potential of Mucorales. Finally, the new methodology employed in transforming *R. microsporus* using the CRISPR-Cas technology will likely be exported to other Mucorales, opening up a range of possibilities for future genetic studies in these ancient fungi.

## Data Availability

Not applicable.
